# Complete genome sequence of Mycobacteriophage Eaglepride

**DOI:** 10.1128/mra.00599-24

**Published:** 2024-09-30

**Authors:** Umar Sahi, Niati G. Kottury, Nirav R. Kottury, Hari Kotturi

**Affiliations:** 1 Department of Biology, University of Central Oklahoma, Edmond, Oklahoma, USA; 2 Virginia Commonwealth University, Richmond, Virginia, USA; Montana State University, Bozeman, Montana, USA

**Keywords:** mycobacteriophages, Eaglepride

## Abstract

Eaglepride is a mycobacteriophage isolated using *Mycobacterium smegmatis* mc^2^155. The genome length is 50,926 bp with 89 open reading frames and 1 tRNA gene. Based on gene content similarity to actinobacteriophages, Eaglepride is assigned to phage subcluster A10 and shares the highest nucleotide identity with mycobacteriophage OKCentral2016.

## ANNOUNCEMENT

The *Mycobacterium* genus contains over 188 species of mycobacteria that are found in a wide array of environments, including garden beds, household plumbing, water samples, and a wide variety of soil types (1, 2). Mycobacteriophages are phages that infect mycobacteria.

**Fig 1:**
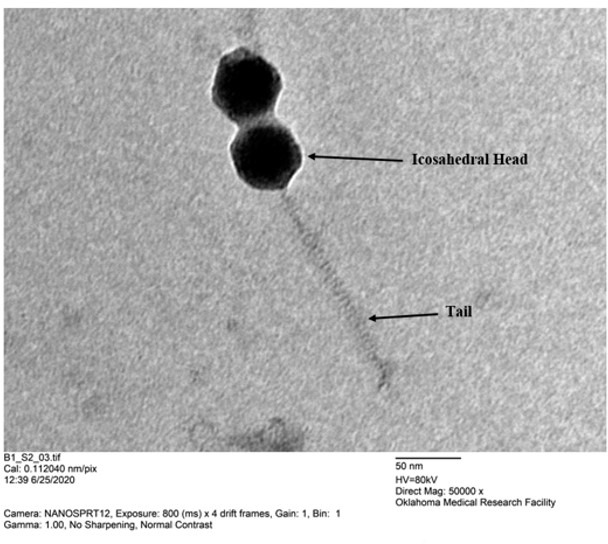
Transmission electron microscopy of Eaglepride on a Formvar-coated copper grid stained with uranyl acetate imaged using Hitachi H-7600 machine.

Here, we report on mycobacteriophage Eaglepride, which was isolated from soil collected in Ashburn, VA (39.014667 N, 77.515417 W), using *Mycobacterium smegmatis* mc^2^155 as the host. Briefly, Eaglepride was extracted by washing the soil with 7H9 liquid medium and collecting the wash through centrifugation and filtration (0.22 µm pore size). The filtrate was then inoculated with *M. smegmatis* mc^2^155 to enrich for mycobacteriophages. After incubation with shaking for 48 hours at 37°C, the enrichment was filtered, plated in 0.8% top agar containing host, and plates incubated at 37°C for 2 days. Eaglepride, which forms clear plaques that are approximately 4 mm in diameter, was plaque-purified through three rounds of purification. Negative-stain transmission electron microscopy revealed Eaglepride to have a Siphovirus morphology and belongs to class Caudoviricetes. The capsid diameter was approximately 55 nm, and the tail length was about 175 nm (Fig. 1, *n* = 3).

The phage genomic DNA was isolated with the Promega Wizard DNA cleanup kit, then processed using an NEB Ultra II FS kit with dual-indexed barcoding to generate sequencing libraries. Sequencing was done on an Illumina MiSeq system using the prepared libraries to yield single-end 150-base reads. Newbler v.2.9 and Consed v.29 were used with default parameters for assembling the genome and assessing the quality of the assembly (3). A shotgun coverage of 1,485 was achieved using 523,000 reads with an average read length of 165 bp. The determination of genome termini was done using the previously described methods (3). During the spring of 2021, the assembled phage genome was annotated by students at University of Central Oklahoma (UCO) and high school students in Virginia. The following software was used for annotation: DNAMaster v.5.23.6 (http://cobamide2.bio.pitt.edu), GeneMark v3.25 (4), Glimmer v3.02b (5), NCBI BLAST v2.9.0 (6), Starterator v1.2 (http://phages.wustl.edu/starterator), HHpred v3.2.0 (7), ARAGORN v1.2.41 (8), Phamerator (9), TMHMM v2.0 (10), and SOSUI v1.11 (11).

The genome length of Eaglepride is 50,926 bp with 64.9% GC content. Eighty-nine open reading frames (ORFs) include one tRNA gene. The genome analysis showed that 43 of the 89 (48.31%) genes within Eaglepride have a known putative function and 46 (58.69%) have no known function. The left arm of the genome contains the structural and assembly genes; the right arm contains genes needed for the lytic cycle. Thirty ORFs are transcribed from left to right, and the rest 54 ORFs are transcribed from right to left. Eaglepride had one programmed ribosomal frameshift, which was associated with the tail assembly chaperone gene. The frameshift occurred at the 15,266 bp and was designated as a −1 frameshift. A tRNA was found within the Eaglepride genome and identified as Pro(cca). The genome also contains a total of three Orphams (ORFs 4, 5, and 72), which are phams containing only a single member. The phage belongs to Cluster A and subcluster A10, sharing 93% nucleotide identity with another mycobacteriophage OKCentral2016 (MF773750), which was determined using NCBI BLAST (6).

## Data Availability

The complete genome sequence of phage Eaglepride is available in GenBank using accession number MZ322017 with NCBI SRA accession number SRX16347219.
